# Inter‐Shot Motion Correction of Segmented 3D‐GRASE ASL Perfusion Imaging With Self‐Navigation and CAIPI

**DOI:** 10.1002/mrm.70437

**Published:** 2026-05-24

**Authors:** Minhao Hu, Frederik J. Lange, Peter Jezzard, Joseph G. Woods, Mark Chiew, Thomas W. Okell

**Affiliations:** ^1^ Oxford Centre for Integrative Neuroimaging, FMRIB, Nuffield Department of Clinical Neurosciences University of Oxford Oxford UK; ^2^ Department of Diagnostic, Interventional and Pediatric Radiology (DIPR), Inselspital Bern University Hospital, University of Bern Bern Switzerland; ^3^ Translational Imaging Center (TIC), Swiss Institute for Translational and Entrepreneurial Medicine Bern Switzerland; ^4^ Physical Sciences Sunnybrook Research Institute Toronto Ontario Canada; ^5^ Department of Medical Biophysics University of Toronto Toronto Ontario Canada; ^6^ The Podium Institute for Sports Medicine and Technology, Department of Engineering Science University of Oxford Oxford UK

**Keywords:** 3D segmented readout, arterial spin labeling, CAIPI sampling, perfusion imaging, retrospective motion correction, self‐navigation

## Abstract

**Purpose:**

Segmented 3D Gradient and Spin Echo (GRASE) is commonly used in Arterial Spin Labeling (ASL) perfusion imaging. However, it is vulnerable to inter‐shot motion, leading to subtraction errors that cannot be corrected. We developed a retrospective self‐navigated inter‐shot motion correction method for segmented 3D‐GRASE ASL imaging with Controlled Aliasing in Parallel Imaging (CAIPI).

**Methods:**

Multiple shots, each uniformly covering k‐space at distinct sample locations, allow a self‐navigator image to be reconstructed using SENSE for each shot. Rigid‐body motion estimation across the self‐navigators is incorporated into a motion‐compensated forward model for image reconstruction. To support self‐navigation, two CAIPI‐sampled segmented 3D‐GRASE trajectories ensuring full k‐space coverage were explored for point spread function profiles and g‐factor effects. Our approach was evaluated against conventional inter‐volume registration and a previously proposed method, alignedSENSE. Additionally, we compared tag‐control interleaving strategies to assess the impact on motion robustness in five healthy volunteers with instructed head motion.

**Results:**

With instructed moderate head motion, our method effectively reduced motion artifacts and outperformed conventional inter‐volume correction by 12.3% in Pearson correlation coefficient, 4.5% in Structural Similarity Index Measure, and 40.1% in temporal SNR. It matched alignedSENSE performance while requiring only 20% of the computational time. All evaluated CAIPI sampling variants enabled robust motion correction, although tradeoffs were observed between through‐plane blurring and SNR. The tag‐control (T/C) inner loop acquisition yielded better motion robustness across quantitative metrics.

**Conclusion:**

Self‐navigated inter‐shot motion correction using CAIPI sampling and a T/C inner loop for segmented 3D‐GRASE ASL can improve image quality and motion robustness.

## 1 Introduction

Arterial Spin Labeling (ASL) is a non‐invasive MRI technique for quantifying cerebral blood flow (CBF), which uses magnetically labeled arterial blood water as an endogenous tracer [1, 2]. By eliminating the need for exogenous contrast agents, ASL is particularly suitable for populations with contraindications to contrast‐based agents, such as patients with renal failure. ASL provides quantitative estimates of CBF, allowing more robust comparisons across brain regions, subjects, and in longitudinal studies [3]. Therefore, ASL has been adopted in both clinical and research settings for evaluating cerebral perfusion and detecting perfusion abnormalities associated with various neurological conditions [4]. It has also been applied beyond the brain, for example, in renal and cardiac perfusion imaging [5].

However, in brain ASL, the perfusion signal derived from the subtraction of tag and control images differs by only about 1%–2% of the signal intensity of static tissue [6]. Therefore, SNR is inherently low for ASL and multiple repetitions are required for averaging. It is also highly sensitive to motion, as even small misalignments, including between individual shots within a single volume, can introduce large subtraction errors and result in inaccurate CBF estimation [7]. Single‐shot 3D acquisitions can mitigate motion sensitivity, but the spatial coverage will be limited, or the echo train will be prolonged, leading to T2‐blurring in the through‐plane direction [6, 8]. To address this issue, segmented (multi‐shot) 3D acquisitions, for example, segmented 3D Gradient and Spin Echo [9] (GRASE) or 3D Rapid Imaging with Refocused Echoes (RARE) stack of spirals [10] are evaluated in prior work [11, 12] and recommended in ASL consensus papers [6]. Segmented 3D acquisitions allow shorter echo trains by dividing k‐space into multiple segments, thereby mitigating T2‐blurring while maintaining high SNR efficiency, whole‐brain coverage and compatibility with background suppression. However, segmentation also leads to a multiplication of the total acquisition time and introduces the risk of inter‐shot motion, that is, subject motion occurring between successive segments. Unlike inter‐volume motion artifacts, such *intra‐volume* inter‐shot motion artifacts cannot be corrected using standard image registration methods in conventional ASL post‐processing pipelines [13]. Developing effective inter‐shot motion correction methods is therefore critical for realizing the full potential of segmented 3D ASL acquisitions.

Prospective motion correction methods aim to reduce inter‐volume and inter‐shot motion artifacts by incorporating low‐resolution navigators [14, 15] or external optical tracking systems [16] to adjust imaging parameters or reacquire [17] corrupted data in real time during the scan. While effective, these approaches often require substantial modifications to the pulse sequence or additional hardware and may lead to increased total acquisition time. As a more practical alternative, Tan et al. [18] incorporated 3D‐GRASE with a widely used retrospective motion correction method Periodically Rotated Overlapping ParallEL Lines with Enhanced Reconstruction [19] (PROPELLER). Huber et al. [20] further improved this by jointly estimating motion and geometric distortion. However, these methods suffered from suboptimal SNR efficiency and were limited to 2D motion detection. Highton et al. [21] proposed an interleaved segmented 3D‐GRASE readout where each shot comprised SENSE‐style undersampled k‐space data. They used SENSE [22] for individual shot reconstruction and corrected inter‐shot motion via rigid image registration. However, by applying registration on images rather than incorporating motion parameters into the reconstruction, their approach introduced interpolation artifacts and higher g‐factor noise amplification penalties. Spann et al. [8] proposed a robust single‐shot 3D‐GRASE readout with time‐dependent 2D Controlled Aliasing In Parallel Imaging Results IN Higher Acceleration [23] (CAIPIRINHA) sampling and incorporated spatio‐temporal total generalized variation regularization to suppress motion artifacts. While this offered improved robustness compared to standard segmented 3D‐GRASE, it did not explicitly correct inter‐shot rigid motion.

Earlier work has demonstrated SENSE‐based motion correction frameworks for segmented 2D multi‐shot imaging, including the matrix formulation [24], the augmented SENSE reconstruction [25] and the application to diffusion imaging [26]. AlignedSENSE [27] further extended these ideas to a general joint estimation framework by incorporating motion directly into the forward model and alternating between motion and image updates. It has been widely applied in various sequences and readout schemes [28].

Building upon these foundations, our work adapts the SENSE‐based motion‐corrected reconstruction concept to segmented 3D sampling, enabling self‐navigated motion estimation with low computational complexity.

**FIGURE 1:**
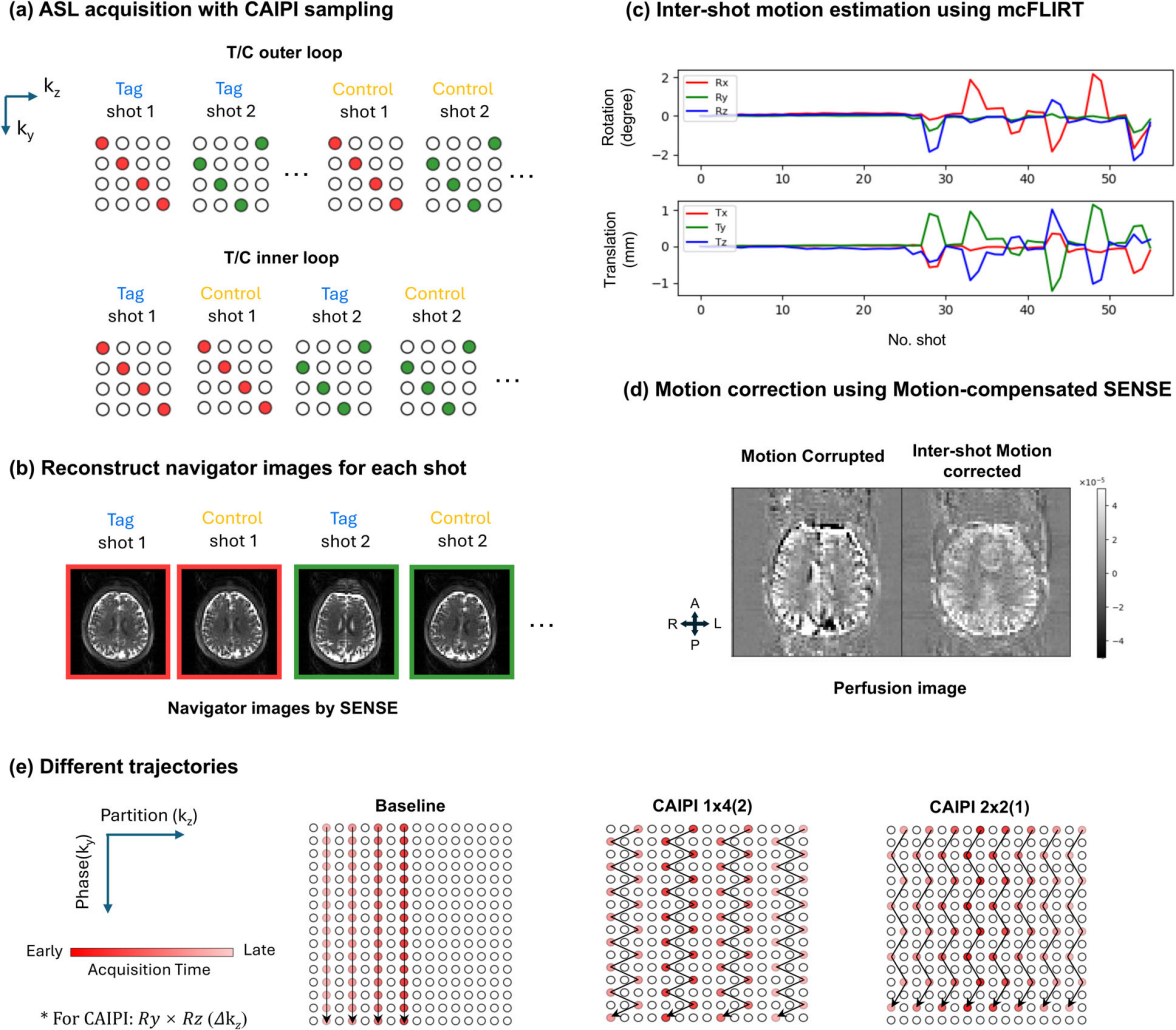
Overview of the proposed approach. (a) The diagram illustrates how segmented k‐space data are acquired across shots over time, with different colors indicating k‐space lines sampled in different shots and under different tag‐control conditions. The T/C inner loop alternates tag and control modes before switching sampling patterns, while the T/C outer loop does so after acquiring each volume. (b) Per‐shot navigator images for tag and control used for motion estimation; residual aliasing may be present. (c) Translation and rotation estimation across shots and tag‐control conditions obtained via mcFLIRT. (d) Reconstruction of motion‐corrected images using a motion‐compensated model. (e) The schematic shows different trajectories including a baseline and two CAIPI variants. Red spots represent the acquired k‐space lines (illustrated for the first shot only), with transparency reflecting acquisition timing (darker means earlier). One solid arrow indicates the trajectory within a single spin echo. For clarity, only a 16 × 16 portion in the phase‐encoding (k*_y_*) and partition (k*_z_*) directions is shown, whereas the actual matrix size is 64 × 32.

In this study, we propose a self‐navigated inter‐shot motion correction method for segmented 3D‐GRASE ASL with Controlled Aliasing in Parallel Imaging (CAIPI) sampling. Figure 1 illustrates the overview of the proposed method. Unlike the approach by Highton et al. [21], the segmented 3D‐GRASE readout employs interleaved CAIPI sampling patterns, with each segment acquired in a single shot. This design reduces the echo train length, thereby mitigating T2 blurring effects. After acquiring all segments, the data can be combined to form a fully sampled k‐space volume, which can be used for sensitivity map estimation. Meanwhile, each individual shot uniformly covers the entire k‐space and so can be reconstructed using SENSE to produce low‐SNR self‐navigator images, which are nonetheless sufficient for motion estimation via image registration. The estimated inter‐shot rigid motion is then incorporated into a final motion‐compensated reconstruction, enabling recovery of a high‐quality motion‐free image from the multi‐shot data without noise amplification or interpolation penalties.

We note that related navigator‐based segmented 3D motion‐correction framework [29], including CAIPI‐style sampling designs, has been reported previously. Compared with this navigator‐based approach [29], our framework is self‐navigated in the sense that motion information is derived directly from the imaging data and incorporated into the motion‐compensated reconstruction. We also note that Liu et al. [29] additionally model secondary motion effects (e.g., field and sensitivity changes), which are not explicitly addressed here.

Using in vivo data from five healthy volunteers, we demonstrated that this method outperformed baseline segmented 3D‐GRASE in the presence of inter‐shot motion. Two CAIPI sampling strategies were evaluated against a baseline trajectory, and three motion correction approaches were compared both quantitatively and qualitatively. Additionally, we investigated the impact of two different tag‐control interleaving schemes to establish whether placing the tag‐control interleaving in the innermost loop was more motion‐robust in practice.

## 2 Methods

### 2.1 Trajectory Design

Some conventional implementations acquire only partial k‐space per shot, such as center‐out half‐partition sampling. While such designs preserve the point spread function (PSF) and reduce T2‐related blurring, the incomplete k‐space coverage per shot limits the feasibility of reconstructing reliable self‐navigator images. Alternatively, other implementations use SENSE‐style undersampling patterns which provide the potential for navigator reconstruction but suffer from higher g‐factor penalties and reduced SNR efficiency [21].

To address this limitation, we propose integrating CAIPI‐style sampling into the segmented 3D‐GRASE acquisition. This produces uniform undersampling patterns characterized by $\mathrm{Ry}\times \mathrm{Rz}\;\left(\Delta k_{z}\right)$, where Ry and Rz are acceleration factors in the in‐plane and through‐plane phase encoding directions, respectively, and $\Delta k_{z}$ is the partition shift applied between successive k‐space lines [23]. By shifting the CAIPI pattern across shots, full k‐space coverage can be achieved after R=Ry×Rz repetitions, allowing both high‐quality final reconstruction and low‐SNR navigator reconstruction from individual shots.

To ensure compatibility with the 3D‐GRASE readout, which comprised multiple spin echoes per shot (defined by the Turbo factor) and multiple EPI gradient echoes between each spin echo (defined by the EPI factor), the CAIPI sampling was designed such that:

EPIfactor×Turbo factor×R=Ny×Nz

where Ny and Nz were the number of lines acquired across all shots in the phase encoding (ky) and partition encoding (kz, i.e., the second phase or slice‐encoding) directions, respectively. In this study, we set the matrix size to Ny=64 and Nz=32, with an effective acceleration factor for each shot of R=4. As a baseline (Figure 1e), we adopted a segmented 3D‐GRASE trajectory, in which we acquired one full partition per spin echo and one phase‐encoding line per EPI gradient echo, that is, an EPI factor of Ny=64 and a Turbo factor of Nz/R=8. In this configuration, each shot sampled every other partition in half of 3D k‐space.

Based on these parameters and constraints described above, we designed and evaluated two CAIPI sampling variants. Detailed acquisition parameters for each strategy are listed in Table 1.

**TABLE 1:** Imaging parameters for the different trajectories.

	Baseline	CAIPI 1 × 4(2)	CAIPI 2 × 2(1)
Turbo factor	8	8	16
EPI Factor	64	64	32
TE (ms)	37.86	39.16	22.52
TR (ms)	3990	4000	4030
Echo spacing (ms)	0.50	0.54	0.52

*Note:* Turbo factor denotes the number of spin echoes per shot, while EPI factor is the number of phase encoding lines within each spin echo. TR indicates the interval between two ASL preparation pulses.

CAIPI1×4(2): Maintained the same EPI and Turbo factors as the baseline trajectory, with eight partitions sampled per spin echo. This design preserved the original echo train length while introducing controlled aliasing along the partition direction.

CAIPI2×2(1): Reduced the EPI factor by half while doubling the Turbo factor to maintain full k‐space coverage. While this resulted in a slightly longer readout time, it potentially improves PSF uniformity and may offer greater robustness to off‐resonance effects due to a larger step size (∆k) in the phase‐encoding direction.

We optimized the sampling to minimize PSF sidelobes and reduced sensitivity to off‐resonance artifacts by ensuring smooth variation of signal intensity and phase accrual across both phase‐encoding and partition directions. In addition to sampling density, the order of k‐space traversal played a critical role in determining the PSF and sensitivity to motion and off‐resonance effects. All proposed trajectories employed symmetric center‐out partition ordering to minimize T2‐weighting and maximize SNR by acquiring central k‐space early in the echo train. Phase‐encoding and partition‐encoding blipped between refocusing pulses were carefully constrained to ensure smooth phase evolution and T2 decay weighting, reducing artifacts that would result from abrupt changes in signal intensity or phase.

PSF simulations were conducted under on‐ and off‐resonance conditions to compare the proposed CAIPI trajectories with the baseline segmented acquisition. The frequency offset of Δf=100Hz was used to approximate typical B_0_ inhomogeneities encountered in 3 T brain imaging near tissue‐air interfaces [30]. The simulations were performed by using MRzero [31], in which the tissue was modeled as a 1D isochromat ensemble (*N* = 64, extent = 4 mm; T1 = 1331 ms [32], T2 = 110 ms [32], T2* = 66 ms [33]). A refocusing flip angle of 120° was used and crusher gradients were applied around each refocusing pulse to suppress unwanted coherence pathways. Effective resolutions (${\text{EffRes}}_{z}$ and ${\text{EffRes}}_{y}$) were quantified as the full width at half maximum (FWHM) of the PSF in each direction. Zero‐padding was applied in k‐space before performing the inverse FFT to obtain better sampling of the PSF. For reference, the ideal effective resolution under T2=∞ conditions would be 100% (normalized to the nominal voxel size). A comparison of different refocusing flip angle schemes is provided in Figure S1.

g‐factor maps were estimated using the pseudo‐multiple replica method [34]. Multiple realizations of Gaussian noise were added to the k‐space data, followed by SENSE [22] reconstruction of both fully sampled and undersampled (*R* = 4) data. Voxelwise g‐factor values were then computed as the ratio of standard deviations across noise realizations between the two datasets, normalized by the square root of the acceleration factor *R*.

### 2.2 Inter‐Shot Motion Correction

The proposed motion correction pipeline consisted of three main stages: navigator image reconstruction, inter‐shot motion estimation, and motion‐compensated image reconstruction. This framework took advantage of the full k‐space coverage enabled by the proposed CAIPI sampling scheme, allowing per‐shot navigator image reconstruction for accurate motion estimation. A total of N pairs of tag and control volumes were acquired, with each volume segmented into R shots so that each shot sampled a unique CAIPI pattern. This ensured that R shots can be combined to form a fully sampled k‐space volume, which was essential for both sensitivity map estimation and motion‐compensated reconstruction. In addition, the use of CAIPI sampling reduced noise amplification by minimizing g‐factor penalties, thereby improving the SNR of both the navigator images and the final motion‐corrected reconstruction.

#### 2.2.1 Navigator Image Reconstruction

Utilizing the full k‐space coverage per shot, each individual shot was reconstructed to generate a self‐navigator image for motion estimation. Unlike joint estimation approaches, such as alignedSENSE, this strategy leveraged the well‐conditioned nature of the CAIPI‐undersampled data to achieve reliable motion estimates through conventional image registration. Both methods can benefit from the g‐factor improvements provided by CAIPI sampling; however, our approach avoids the computational complexity associated with iterative joint optimization methods. Similar CAIPI‐based segmented 3D acquisition designs for navigator‐driven motion correction have been explored previously [29].

For each shot, an undersampled image was reconstructed using SENSE with l2‐regularization.

$${\hat{x}}_{l}=\arg\;\min_{x}{\left\|M_{l}\mathrm{FSx}-y_{l}\right\|}_{2}^{2}+\lambda {\left\|x\right\|}_{2}^{2}$$

where ${\hat{x}}_{l}$ denotes the reconstructed self‐navigator image for the l‐th shot (l∈[1,R]), $y_{l}$ is the corresponding acquired k‐space data, $M_{l}$ is the undersampling mask for l‐th shot, F is the Fourier transform operator and S is the operator for coil sensitivity maps. λ is the Tikhonov regularization parameter and is empirically set to $1\times 10^{-3}$ as this was found to be a good balance between image sharpness and noise (Figure S2). The optimization problem is solved using the Conjugate Gradient (CG) algorithm.

To improve robustness and ensure full k‐space coverage for coil sensitivity estimation, all acquired volumes were temporally averaged before ESPIRiT [35] calibration. We also considered estimating sensitivity maps from a reduced subset of minimally moving shots or volumes (Figure S3); however, using all volumes provided higher SNR and more stable calibration in our data, and we therefore adopted it for fair and consistent comparisons. Although subject motion may introduce inconsistency across shots, ESPIRiT has been shown to exhibit inherent robustness to moderate motion artifacts due to its calibration region design [21]. In addition, we set the crop threshold to zero, ensuring that the estimated sensitivity maps extended across the entire field of view, including peripheral regions. This mitigated sensitivity map truncation under large displacements, which would otherwise introduce discontinuities leading to noise amplification and unstable CG convergence.

#### 2.2.2 Inter‐Shot Motion Estimation

Although minor non‐rigid components may exist, head motion is typically modeled as a rigid‐body transformation which is widely adopted in previous work [36]. The rigid transform has six degrees of freedom (DOF): three translations $\left(t_{x},t_{y},t_{z}\right)$ and three rotations about the three axes $\left(r_{x},r_{y},r_{z}\right)$.

We denote the rigid‐body transformation operator for the l‐th shot as ${\hat{T}}_{l}$, defined by 6‐DOF parameter set ${\left(t_{x},t_{y},t_{z},r_{x},r_{y},r_{z}\right)}_{l}$. These parameters were estimated by registering each navigator image ${\hat{x}}_{l}$ to a reference image $x_{\mathrm{ref}}$, which was chosen as the first shot of the first volume, i.e., $x_{\mathrm{ref}}={\hat{x}}_{1}$. Image registration was performed using mcFLIRT [36], with the cost function set to normalized correlation (normcorr) to improve robustness under low‐SNR conditions. Although each shot corresponds to a different CAIPI interleave, each shot still provides an approximately uniformly distributed undersampling of k‐space; therefore, the reconstructed navigator images primarily differ in SNR and residual aliasing, rather than exhibiting fundamentally different intrinsic contrast. Using normalized correlation further mitigates sensitivity to global intensity scaling and contrast differences between shots, reducing the risk of spurious motion driven by shot‐to‐shot intensity variations. Per‐shot navigator reconstructions for both Tag and Control repetitions are shown in Figure S4, confirming consistent anatomical depiction across all shots with no evidence of shot‐dependent contrast differences.

#### 2.2.3 Motion‐Compensated Reconstruction

The estimated motion parameters are incorporated into a motion‐compensated forward model [24, 25] to reconstruct a motion‐corrected image by solving the following optimization problem:

$$\hat{x}=\arg\;\min_{x}{\left\|\sum_{l=1}^{R}M_{l}\mathrm{FS}{\hat{T}}_{l}x-y_{l}\right\|}_{2}^{2}$$

Inspired by alignedSENSE [27], which avoids explicit regularization, we leveraged the well‐conditioned nature of the motion‐compensated encoding operator, due to parallel coil redundancy and full k‐space coverage, to achieve a stable and accurate reconstruction using a CG optimization. Although each shot is acquired at *R* = 4, the motion‐compensated reconstruction is effectively fully sampled, with only small residual k‐space gaps expected from trajectory shifts such as subject rotations. Accordingly, the encoding remained sufficiently well‐conditioned for stable CG convergence without explicit L2 regularization, as confirmed empirically by the λ‐sweep in Figure S5. For stronger acceleration or lower‐SNR settings, Tikhonov regularization and early stopping may provide additional stabilization. The motion operator applies the per‐shot rigid body transforms in image space using convolution‐based interpolation [37]. The CG solver was run with a maximum of 200 iterations and convergence was typically reached earlier using a relative residual error tolerance of $1\times 10^{-10}.$

In addition to inter‐shot motion, inter‐volume motion can also degrade the final ASL perfusion images. In this study, both inter‐shot and inter‐volume motions were accounted for by aligning the navigator images from all shots and volumes to a single reference. The resulting motion parameters were integrated into the motion‐compensated reconstruction, eliminating the need for extra image registration in post‐processing and avoiding interpolation‐induced blurring.

All reconstructions were implemented in MATLAB R2023a and Python 3.12.8, and executed on a workstation equipped with 2 AMD EPYC 9274F CPU (24 cores) and 24 GB RAM. The average total processing time for one subject, including self‐navigator reconstruction, motion estimation, and motion‐compensated reconstruction, was approximately 45 min.

### 2.3 Tag‐Control Interleaving

As recommended by the ASL consensus papers [6, 38], the tag‐control (T/C) loop should be placed in the innermost loop position when using a segmented readout to ensure accurate subtraction. However, this strategy has not been consistently adopted in all studies in the literature and, to the best of our knowledge, its impact on motion robustness has not been thoroughly evaluated.

In this study, we investigated the effect of T/C interleaving loop order on ASL image quality by comparing two configurations (Figure 1a): (1) T/C inner loop, where a shot with the same CAIPI sampling pattern was acquired for tag and then control conditions before moving on to the next shot; and (2) T/C outer loop, where all tag shots for a volume were acquired before acquiring the control shots.

The T/C inner loop configuration was hypothesized to enhance motion robustness by minimizing the temporal gap between corresponding tag and control shots, thereby reducing misalignment due to motion, particularly under moderate motion conditions.

### 2.4 Data Acquisition

We modified a previously described 3D‐GRASE pseudo‐Continuous ASL (pCASL) sequence [39, 40] on a 3T Prisma scanner (Siemens Healthineers, Erlangen, Germany) equipped with a 32‐channel receive‐only head coil. The sequence included Water suppression Enhanced through T1 effects (WET) [41] presaturation and two global background suppression inversion pulses with timing optimized to null tissues with T1 equal to 700 or 1400 ms at a time point 100 ms prior to readout excitation. Simulations indicated residual signals of approximately 7%–9% across tissues at excitation, providing strong GM/WM suppression while preserving sufficient static tissue signal for motion estimation.

The pCASL parameters were: tag RF flip angle =20°, duration =500μs, mean tag gradient =0.80mT/m, tag gradient amplitude =6.0mT/m, labeling duration (LD) =1800ms, post‐label delay (PLD) =1800ms, ${\mathrm{TR}}_{\mathrm{ASL}}=4000\;\mathrm{ms}$ (time between ASL preparations). The labeling plane was positioned through the proximal V3 segment of the vertebral arteries as per previous studies [42].

3D‐GRASE readout parameters were: refocusing flip angle =120°, FOV $=230\times 230\times 115\;{\mathrm{mm}}^{3}$, matrix size =64×64×32, voxel size =3.6mm isotropic, bandwidth =2298Hz/pixel, and no slice oversampling. Echo shifting was applied for all trajectories, where applicable, to smooth the signal evolution across shots. Additional readout parameters are summarized in Table 1.

In each session, a T1‐weighted anatomical image (1.7mm isotropic) was acquired for registration and tissue segmentation (FOV $=220\times 200\times 218{\mathrm{mm}}^{3}$, TR/TE/TI=1900/3.71/904ms, flip angle =8°, acquisition time =3m40s).

Five healthy volunteers (1 female, age 26±1.7 years) were scanned under a technical development protocol approved by local ethics and institutional committees. Subjects remained stationary during the acquisition of six motion‐free 3D‐GRASE volumes (three tag‐control pairs; total time = 1 min 36 s) followed by moderate cued head motion during the acquisition of eight additional volumes (four tag‐control pairs; total time = 2 min 8 s). The motion was guided by a visual stimulus [43] to ensure consistency across scans. The instructed motion consisted of nodding and head rotations performed at a moderate, continuous pace every 15 s during the acquisitions. The total acquisition time for seven tag‐control pairs was 3 min 44 s.

Acquisitions with three different k‐space trajectories were tested in each subject, including one 3D‐GRASE baseline and two CAIPI sampling variants (see Table 1 for details). Both CAIPI experiments were repeated with two tag‐control interleaving strategies: T/C inner loop and T/C outer loop. The baseline trajectory used only the T/C outer loop.

### 2.5 Evaluation

Prior to computing quantitative metrics, all 14 volumes (seven tag‐control pairs), including the motion‐free acquisitions, were first processed using the same reconstruction pipeline and then registered to the midpoint of the first six volumes using FLIRT (6‐DOF) together with FSL's *midtrans* and *applywarp* utilities, ensuring consistent interpolation blurring. This yielded seven aligned tag‐control pairs. Perfusion‐weighted images were then generated by pairwise subtraction for each aligned tag‐control pair. A brain mask and a gray matter mask were generated from the T1‐weighted image using FSL tools [44] and then registered to the ASL volumes.

Temporal SNR (tSNR) was computed voxel‐wise across the seven aligned perfusion volumes and averaged within the gray‐matter mask.

For similarity evaluation, the first three motion‐free volumes were first motion‐corrected then averaged to create a reference image $I_{r}$, while the mean of all seven motion‐corrected volumes was used as the test image $I_{t}$. Pearson correlation coefficient (PCC) was calculated between $I_{r}$ and $I_{t}$ after applying the brain mask and flattening both into voxel‐wise vectors. Structural similarity index (SSIM) was computed using a Gaussian kernel with sigma = 1.5 and kernel size of 11 voxels isotropic, with constants k1 = 0.01 and k2 = 0.03, and then averaged across all voxels within the brain mask to obtain a single score.

To assess the statistical significance of any improvements achieved by motion correction, paired *t*‐tests were conducted across subjects for all metrics when comparing different methods. A *p* value lower than 0.05 was considered statistically significant. In addition to the paired *t*‐tests, a multi‐way ANOVA was performed to assess the combined effects of acquisition trajectory, tag‐control interleaving strategy, and motion‐correction method on the quantitative metrics.

For comparison, alignedSENSE reconstructions were implemented using the same CG framework and stopping criteria as used in our method. Iterations were terminated upon convergence of the relative residual (1 × 10^−10^) or after a maximum of 200 iterations, whichever occurred first. In practice, motion‐free or mildly motion‐corrupted data typically converged before reaching the iteration limit, whereas severely motion‐corrupted cases occasionally required the full 200 iterations.

## 3 Results

### 3.1 Trajectory Simulations

**FIGURE 2:**
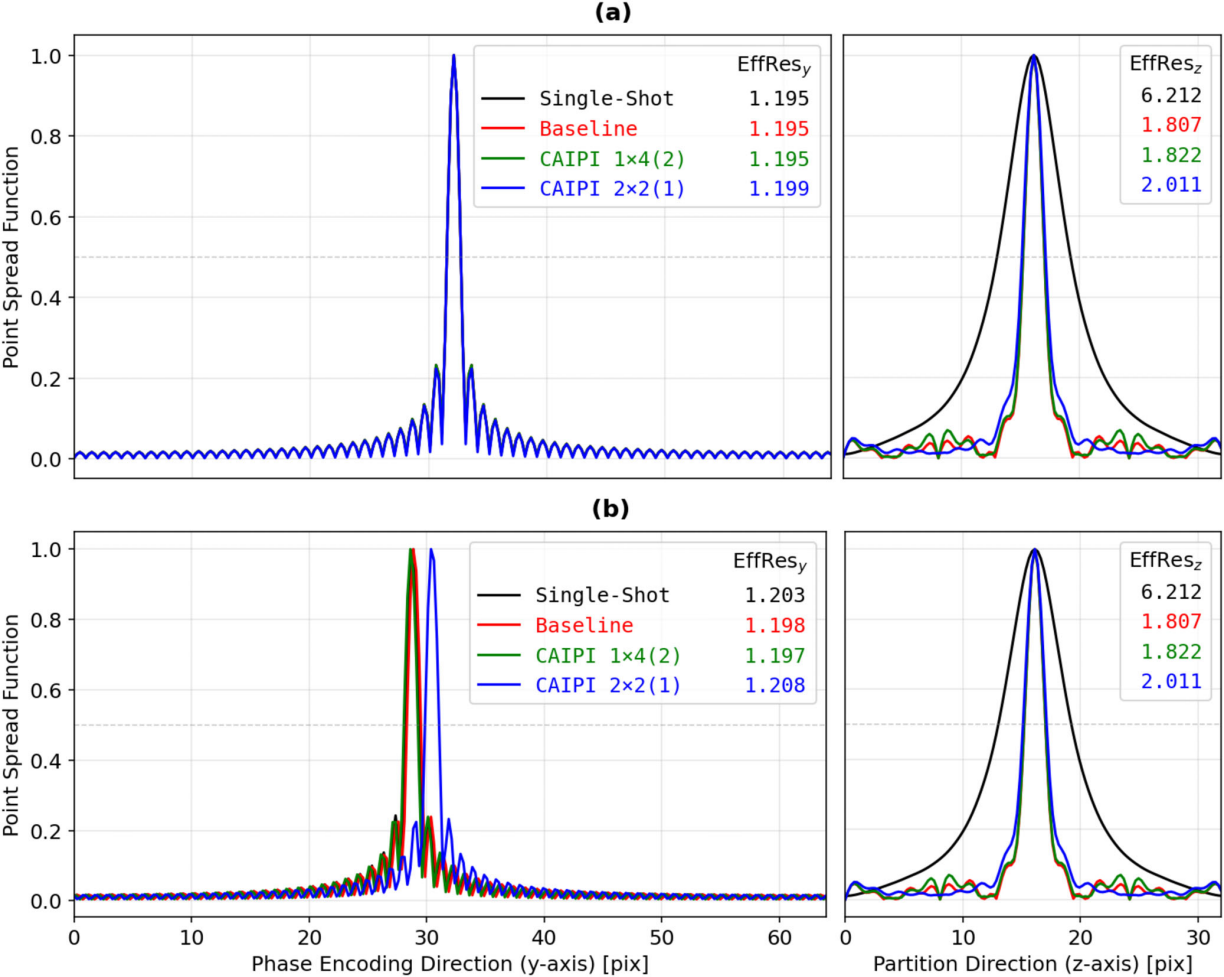
Simulated point spread function (PSF) of different acquisition trajectories along the two phase‐encoding directions. (a) PSF for on‐resonance, and (b) PSF for off‐resonance with a simulated frequency offset of 100 Hz. The single‐shot PSF is included for comparison, demonstrating that segmented methods yield improved performance. The effective resolution was determined by measuring the full width at half maximum (FWHM) of the main lobe of the PSF in simulation. Effective resolution along each axis (${\text{EffRes}}_{z}$ and ${\text{EffRes}}_{y}$) is indicated in the corresponding colors for each trajectory. The simulations were performed using T1 = 1331 ms, T2 = 110 ms, T2* = 66 ms and a refocusing flip angle of 120°. Note that to compute the FWHM, the k‐space data were zero‐padded before applying the inverse FFT, and the FWHM of the main lobe was measured.

To evaluate the effective resolution and noise amplification of the proposed k‐space sampling strategies, we simulated the PSF and computed g‐factor maps for comparison. Figure 2 illustrates the PSF profiles along the phase‐encoding direction (y‐axis) and partition direction (z‐axis) under both on‐resonance (Figure 2a) and 100Hz off‐resonance (Figure 2b) conditions.

In the on‐resonance setting, the baseline trajectory exhibited the narrowest effective resolution in the partition direction $\left({\text{EffRes}}_{z}=180.7\%\right)$ of ideal, followed closely by CAIPI1×4(2) (182.2%). CAIPI2×2(1) showed slightly broader resolution (201.1%), while the single‐shot trajectory exhibited substantial blurring (621.2%) due to its longer echo train. Along the phase‐encoding direction, the effective resolution was similar across all designs (${\text{EffRes}}_{y}=119.5\%$), with CAIPI2×2(1) showing only a marginal increase (119.9%). Under 100 Hz off‐resonance conditions, ${\text{EffRes}}_{z}$ remained unchanged while ${\text{EffRes}}_{y}$ slightly increased.

**FIGURE 3:**
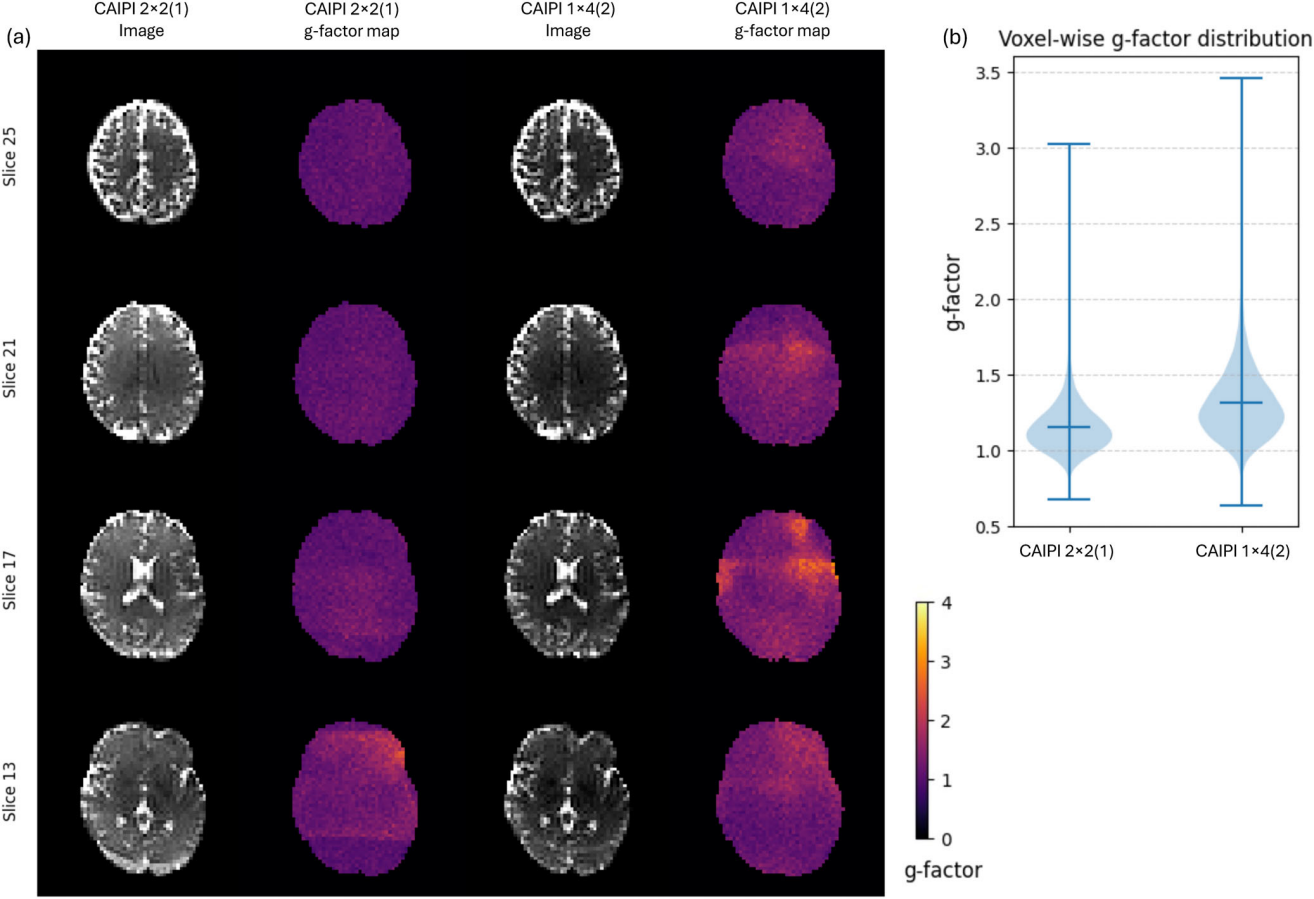
Comparison of navigator image g‐factor maps and distributions for different CAIPI sampling trajectories. (a) Representative navigator axial slices (from superior to inferior) show g‐factor maps for CAIPI 2 × 2(1) and CAIPI 1 × 4(2) sampling patterns. The CAIPI 2 × 2(1) pattern demonstrates more uniform g‐factor distributions with lower values in central brain regions (dark purple areas) while the CAIPI 1 × 4(2) pattern exhibits higher overall g‐factors with a more heterogeneous spatial distribution (red and yellow areas). (b) Violin plot of voxel‐wise g‐factors from subject 2, confirming the better noise performance and greater uniformity achieved with CAIPI 2 × 2(1) sampling pattern.

Both of the CAIPI variants were able to successfully reconstruct good quality navigator images in vivo, although with differing noise amplification properties. g‐factor maps (Figure 3) for the reconstruction of the navigator images were compared between the two CAIPI variants to assess noise amplification due to undersampling within the parallel imaging reconstruction. The CAIPI2×2(1) pattern exhibited lower g‐factors overall (mean = 1.15 within brain mask) with better values in central regions but elevated values near the edges, especially in inferior brain regions. The CAIPI1×4(2) trajectory, in contrast, showed higher overall noise amplification (mean = 1.32 within the brain mask) and demonstrated a wider distribution of g‐factor values with less spatial homogeneity.

### 3.2 Comparison of Different Inter‐Shot Motion Correction Methods

**FIGURE 4:**
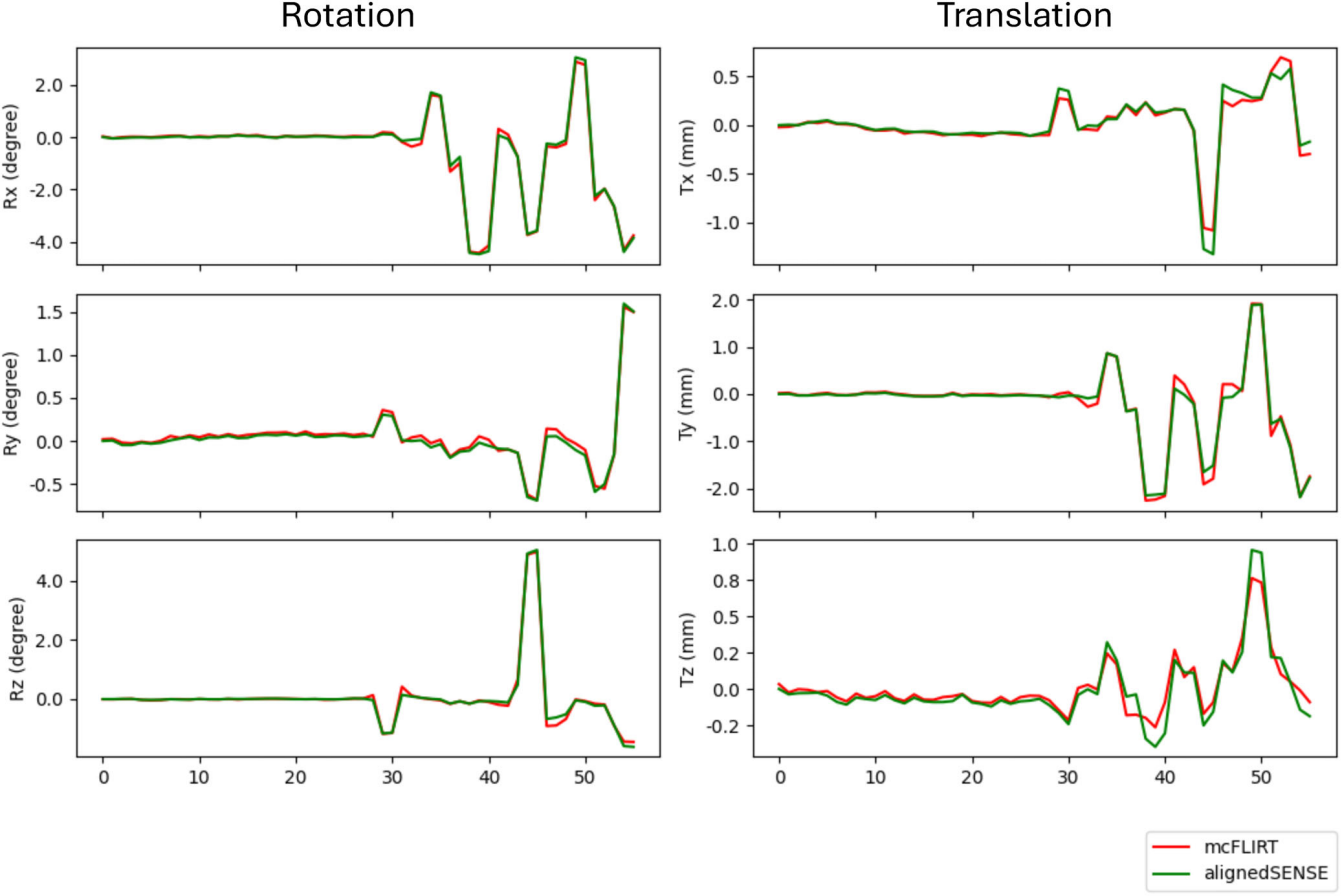
Comparison of motion parameters estimated by mcFLIRT applied to the navigator images (red) and alignedSENSE (green) for subject 4 with the CAIPI 1 × 4(2) trajectory and T/C inner loop. The rotation center is set to the origin of the coordinate system.

Figure 4 shows the estimated rigid‐body motion parameters for one representative subject under the T/C inner loop acquisition condition, comparing results from image registration of navigator images using mcFLIRT and alignedSENSE [27] joint estimation. The horizontal axis represents the cumulative shot index, with each complete volume comprising four shots (R=4). The subject remained still during the first 24 shots (six volumes, three tag‐control pairs), followed by cued moderate head movements. Both methods produced similar motion estimates, with only minor differences observed relative to the overall motion amplitude, indicating consistent and reliable motion estimation across frameworks.

Table 2 presents the quantitative metrics (PCC, SSIM and tSNR) of the final corrected images, comparing the proposed inter‐shot motion correction method versus alignedSENSE. The results were averaged across all five subjects, all trajectories (CAIPI2×2(1) and CAIPI1×4(2)), and both T/C interleaving strategies. The proposed inter‐shot motion correction method demonstrated comparable performance to alignedSENSE across all metrics, with a modest improvement observed in the mean values of PCC (0.79 vs. 0.78), SSIM (0.77 vs. 0.76) and tSNR (2.02 vs. 1.91). Paired *t*‐tests indicated that the improvement in tSNR was statistically significant (*p* = 0.02), whereas the differences in PCC (*p* = 0.43) and SSIM (*p* = 0.71) were not statistically significant. The proposed method achieved these results with substantially reduced computational time, approximately five times faster than alignedSENSE due to the elimination of iterative joint optimization. Results separated by T/C interleaving condition are provided in Table S1.

**TABLE 2:** PCC, SSIM, and tSNR values for different motion estimation and correction methods.

Methods	PCC	SSIM	tSNR
alignedSENSE	0.78 ± 0.13; 0.81 [0.52–0.95]	0.76 ± 0.10; 0.76 [0.59–0.91]	1.91 ± 0.80; 1.72 [0.76–4.22]
Self‐navigation + mcFLIRT (proposed method)	0.79 ± 0.10; 0.79 [0.62–0.96]	0.77 ± 0.09; 0.77 [0.62–0.91]	2.02 ± 0.87; 1.91 [0.79–4.29]

*Note:* The results are averaged across all five subjects, all trajectories (baseline, CAIPI 2 × 2(1) and CAIPI 1 × 4(2)) and both tag‐control interleaving strategies. Values are reported as mean ± standard deviation; median [min—max] across subjects.

**FIGURE 5:**
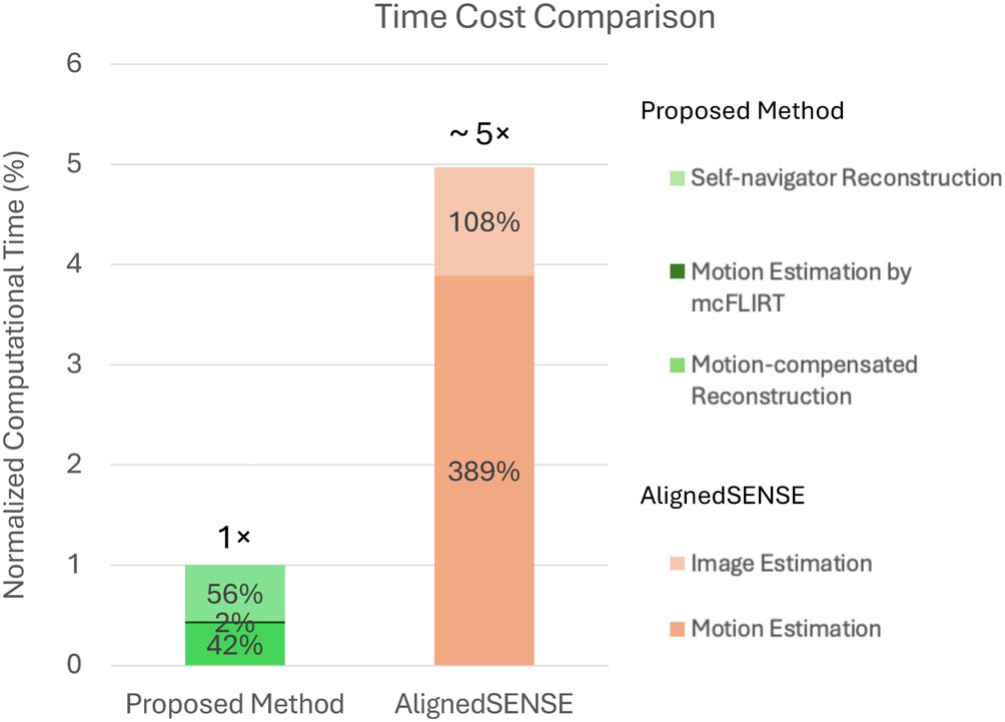
Computational time comparison between the proposed method and alignedSENSE on the same hardware. The bar chart shows the normalized computation time for different processing components, with the proposed method set as the reference (1×). The proposed method consists of three main stages: Self‐navigator reconstruction (56% of total time), motion estimation by mcFLIRT (2% of total time), and motion‐compensated reconstruction (42% of total time). alignedSENSE requires about 5× longer processing time, with the majority spent on iterative joint image and motion estimation (108% and 389% of proposed method's total time respectively).

Figure 5 presents the computational time breakdown for both methods. The proposed approach required only 20% of the total processing time compared to alignedSENSE. The efficiency stems from the straightforward pipeline: self‐navigator reconstruction (56%), motion estimation via mcFLIRT (2%), and motion‐compensated reconstruction (42%). In contrast, alignedSENSE's iterative joint optimization dominated processing time, consuming 389% of the proposed method's total time for motion estimation and 108% for image estimation.

### 3.3 Comparison of Different Trajectories and T/C Interleaving Strategies

**FIGURE 6:**
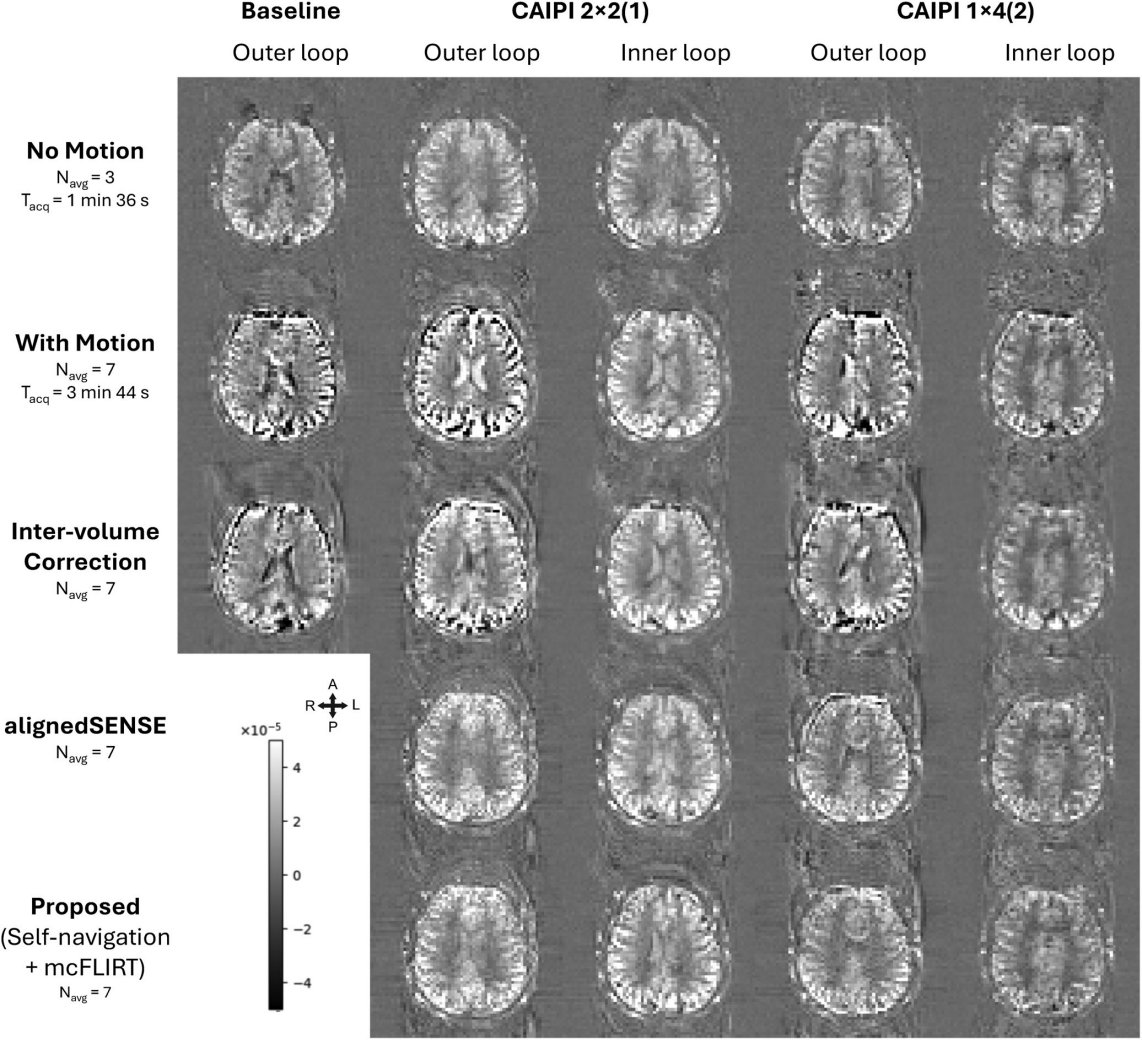
Visualization of perfusion images for subject 4 (axial view). The perfusion images are generated by subtracting the magnitude of the tag images from the control images and averaging over time. Both positive and negative signals are shown here to highlight the appearance of artifacts. “No Motion” indicates the perfusion images obtained when the subject remained still (three tag‐control pairs), while “With Motion” shows the averaged images from the whole experiment, including four tag‐control pairs captured when the subject moved their head according to instructions. “Inter‐volume Correction” refers to post‐processing image registration across volumes, whereas “alignedSENSE” and “Proposed” describe the alignedSENSE method and the proposed method that addresses inter‐shot motion. Note that the proposed inter‐shot motion correction cannot be performed using the baseline trajectory due to the highly asymmetric k‐space sampling within each shot. The results demonstrate that the proposed inter‐shot correction, especially when combined with T/C inner loop interleaving, substantially reduces motion‐related artifacts and improves perfusion image quality.

**FIGURE 7:**
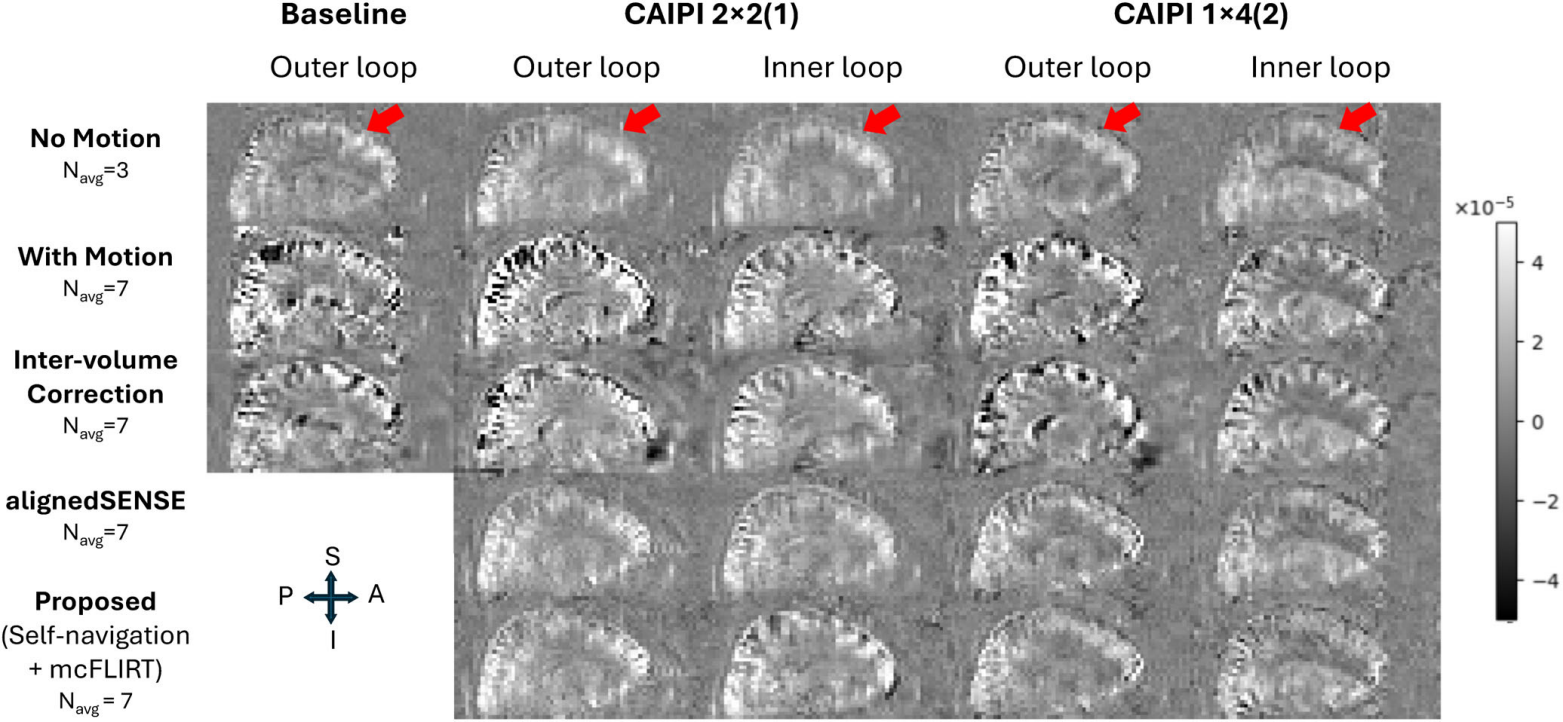
Visualization of perfusion images for subject 4 (sagittal view). Arranged as per Figure 6. Blurring along the partition (kz) direction is highlighted with red arrows, most apparent for the CAIPI 2 × 2(1) trajectory due to the longer echo train. In contrast, CAIPI 1 × 4(2) preserves resolution comparable to the baseline trajectory, while remaining applicable to the proposed inter‐shot motion correction.

Figures 6 and 7 present the temporally averaged perfusion images of Subject 4 in the axial and sagittal planes, respectively, comparing motion‐free reference images, motion‐corrupted images, and results after inter‐volume and inter‐shot motion correction across different trajectories and T/C interleaving strategies. Comparing the motion‐free and motion‐corrupted images revealed that inter‐shot motion introduced strong artifacts, particularly near gray matter‐CSF boundaries. Inter‐volume motion correction mitigated these artifacts somewhat, but residual artifacts remained evident. In contrast, the proposed inter‐shot motion correction greatly reduced the impact of these artifacts, restoring perfusion signals closer to the motion‐free reference. An additional subject is shown in Figure S1.

Regarding T/C interleaving, the outer loop consistently exhibited more severe motion artifacts, especially near the brain edges, and the motion‐corrected images were noisier compared to using T/C inner loop. Among the trajectories, CAIPI2×2(1) was considerably blurrier in the sagittal view (Figure 7) due to its longer echo train.

**FIGURE 8:**
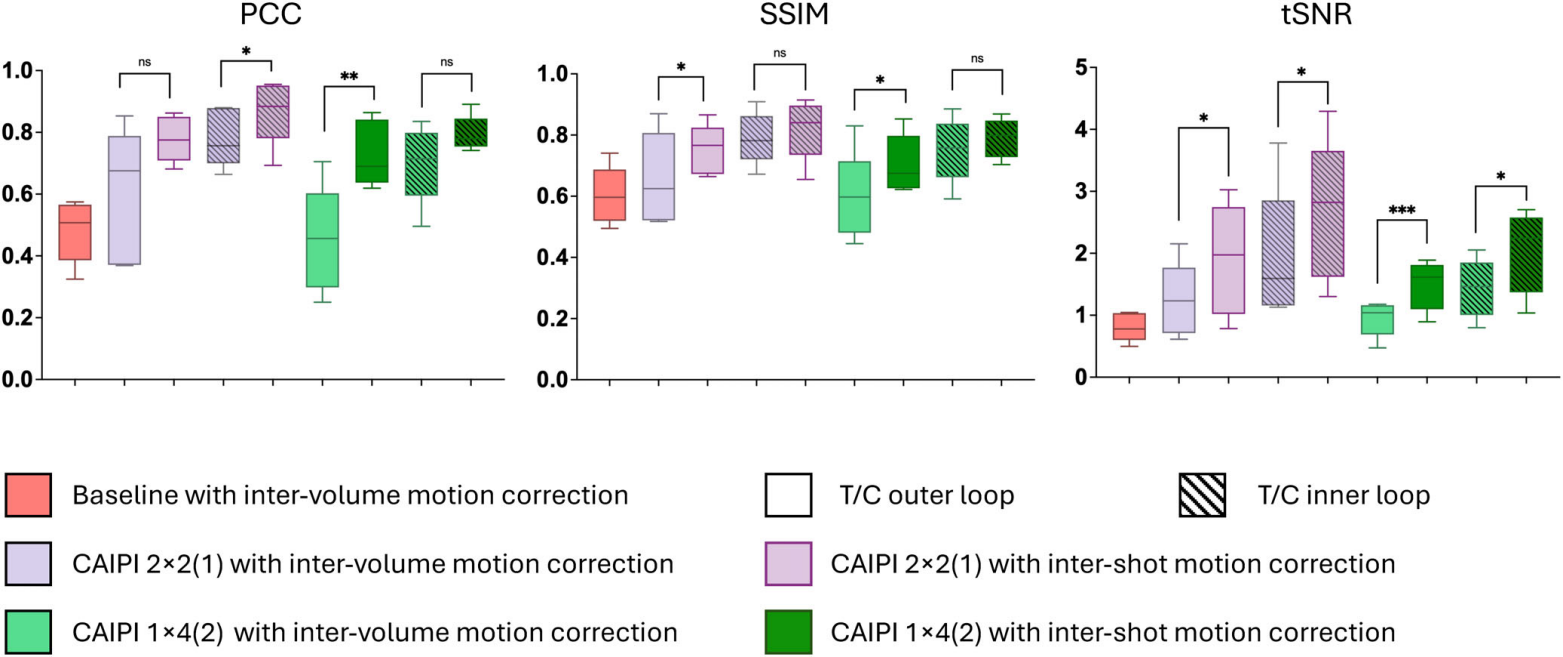
Quantitative comparison of inter‐volume motion correction and the proposed inter‐shot motion correction across various metrics, including PCC, SSIM, and tSNR for all trajectories. A paired *t*‐test was conducted to compare values before and after motion correction. Significance levels: ns (*p* > 0.05), *(*p* ≤ 0.05), **(*p* ≤ 0.01), ***(*p* ≤ 0.001).

Figure 8 quantitatively compares inter‐volume and inter‐shot motion correction across all trajectories in five subjects using PCC, SSIM, and tSNR metrics. Results were grouped by sampling trajectory (Baseline, CAIPI2×2(1), CAIPI1×4(2)) and T/C interleaving strategy (outer loop vs. inner loop). Across all CAIPI trajectories with T/C inner loop, the proposed inter‐shot motion correction method consistently outperformed inter‐volume correction. On average, the inter‐shot correction method improved PCC by 12.3%, SSIM by 4.5%, and tSNR by 40.1% compared to inter‐volume correction. Although the metric differences did not always reach significance, a consistent trend favoring inter‐shot correction was observed across all settings, including T/C outer loop.

Comparing inner vs. outer T/C loop configurations within each trajectory showed that the T/C inner loop consistently yielded better image quality and motion correction performance. For CAIPI2×2(1), the inner loop improved performance by 20.0% for PCC, 14.3% for SSIM and 46.4% for tSNR compared to the outer loop. Similarly, CAIPI1×4(2) showed improvements of 26.7% for PCC, 18.4% for SSIM and 42.2% for tSNR. These results highlighted the advantage of acquiring T/C pairs in close temporal proximity to minimize interleaving‐related motion effects.

The baseline trajectory, which used a T/C outer loop and did not support inter‐shot correction due to half k‐space coverage per shot, exhibited the lowest performance across all metrics (PCC: 0.48, SSIM: 0.60 and tSNR: 0.81). In contrast, both CAIPI2×2(1) and CAIPI1×4(2) trajectories achieved substantially higher scores, even with inter‐volume correction only. Notably, CAIPI2×2(1) with inter‐shot correction and T/C inner loop delivered the best overall performance (PCC: 0.87, SSIM: 0.82, tSNR: 2.67).

To further assess the statistical significance of the observed differences, a multi‐way ANOVA was conducted on the combined metrics across all CAIPI trajectories (excluding baseline trajectory), with factors including trajectory type, T/C interleaving strategy, and motion correction method. T/C interleaving strategy showed an extremely significant effect (*F* = 28.58, *p* < 0.001), providing strong statistical evidence that the inner loop configuration consistently outperforms the outer loop approach. Trajectory type also demonstrated a significant effect (*F* = 10.24, *p* = 0.003), confirming meaningful performance differences between the two CAIPI sampling variants. Finally, the choice of motion correction method showed a significant effect (*F* = 33.58, *p* < 0.001), confirming the benefits of inter‐shot correction over conventional inter‐volume methods.

## 4 Discussion

In this study, we proposed a self‐navigated inter‐shot motion correction framework for segmented 3D‐GRASE ASL imaging, enabled by CAIPI sampling trajectories. Our results demonstrated that this approach improved motion robustness and overall image quality compared to a conventional inter‐volume correction method and achieved comparable performance to joint estimation methods such as alignedSENSE, using fewer computational resources. In addition, we evaluated the impact of tag‐control interleaving strategies, providing experimental evidence supporting the use of an innermost T/C loop in segmented 3D acquisitions.

The use of CAIPI sampling played a central role in the effectiveness of our method. CAIPI sampling enabled each shot to be reconstructed individually with sufficient image quality for reliable motion estimation, whereas the baseline sampling pattern (i.e., half k‐space coverage per shot) did not provide adequate data for robust per‐shot reconstruction. Although the additional phase blips required in both y and z directions for CAIPI slightly compromise the PSF, CAIPI 1 × 4(2) still demonstrated excellent resolution under on‐ and off‐resonance conditions while CAIPI 2 × 2(1) exhibited more blurring in the partition direction due to the larger number of refocusing pulses required, which can be observed in Figure 7.

The proposed motion correction pipeline leveraged the full k‐space coverage of CAIPI sampling to reconstruct per‐shot navigator images without the need for a separate navigator acquisition, which would add acquisition complexity, perturb the magnetization evolution and usually result in lower‐resolution navigator images [45]. Our approach allowed accurate rigid‐body motion estimation for each shot using conventional registration tools, for example, mcFLIRT [36]. This was an advantage over conventional inter‐volume registration performed in post‐processing, which cannot correct for inter‐shot motion, whilst also avoiding potential interpolation‐induced blurring in post‐processing by incorporating motion correction directly into the reconstruction. Our experimental results demonstrated that inter‐shot motion correction consistently improved PCC, SSIM, and tSNR metrics across all tested trajectories, particularly when used in combination with an innermost T/C loop.

The approach of reconstructing individual shots for image‐based navigation has been investigated in previous work for various acquisition schemes [46, 47]. However, the key insight here was that the CAIPI sampling design enabled a simpler implementation than joint estimation methods like alignedSENSE. While alignedSENSE provides a powerful and general framework capable of handling cases where individual shots cannot be reliably reconstructed, our method achieves comparable performance through a simpler single‐step reconstruction that uses fixed motion estimates derived from image registration. For the specific case of CAIPI sampling with moderate acceleration (*R* = 4), the well‐conditioned per‐shot reconstruction quality eliminated the need for complex joint simultaneous motion estimation by aligning all navigator images to a single reference, requiring no additional post‐processing stages while achieving similar image quality with substantially reduced computational time.

The order of the tag‐control interleaving loop also had a significant impact on motion robustness. While consensus guidelines have recommended the use of an inner loop configuration, our study provided supporting evidence for this recommendation in the context of segmented 3D‐GRASE. We observed that inner loop T/C pairing consistently reduced motion artifacts and yielded cleaner perfusion maps, especially in cortical regions where motion sensitivity is highest. We also verified that the choice of reference image for motion estimation (e.g., label, control, or mean tag‐control images) had no noticeable impact on the estimated motion parameters, likely because static tissue signal remained prominent under the moderate background suppression used in this study. More generally, the degree of background suppression represents a trade‐off between reducing static tissue contamination and preserving sufficient SNR for reliable shot‐wise motion estimation, and exploring alternative strategies such as operating on shot‐level subtraction images may be an interesting direction for future work.

Despite promising results, this study still has several limitations. First, all experiments were conducted on healthy volunteers with instructed moderate motion. In practice, motion patterns can vary significantly between individuals. Further validation in patient populations and under more natural, uncontrolled motion conditions is necessary. Second, the acceleration factor of each navigator used in this study was limited to *R* = 4, which allowed high‐quality self‐navigator reconstruction. At higher acceleration factors, the increased noise propagation may reduce the reliability of shot‐wise motion estimation in self‐navigated reconstructions. In such cases, iterative joint estimation frameworks like alignedSENSE could offer greater robustness at the cost of additional computation, as similarly discussed in Steinhoff et al. [48]. Third, the current implementation requires offline reconstruction processing. While the computational efficiency of the proposed method makes real‐time implementation more feasible than iterative approaches like alignedSENSE, integration into scanner console software would require further development to enable online motion correction. Fourth, we acknowledge that there are many feasible combinations of protocol parameters. Alternative settings such as higher turbo factors or fewer repetitions may provide different trade‐offs among scan time, through‐plane blurring and potentially motion robustness. In this study, we evaluated only a limited set of configurations to maintain evenly distributed CAIPI sampling patterns and to avoid substantial changes to the shot‐to‐shot trajectories. Future work will further explore protocol optimization.

The principles underlying this approach are consistent with previously reported SENSE‐based motion correction frameworks that have been applied to other multi‐shot sequences such as segmented EPI [26, 46, 47]. Our contribution extends these concepts to 3D‐GRASE acquisitions, demonstrating the feasibility of self‐navigated inter‐shot motion correction in 3D. Further research is warranted to evaluate the framework under higher acceleration factors, more complex motion patterns, and potential inline implementations.

## 5 Conclusions

We have presented a self‐navigated inter‐shot motion correction framework for segmented 3D‐GRASE ASL enabled by CAIPI sampling, which allows robust per‐shot navigator reconstruction and accurate rigid motion estimation. This approach effectively mitigates inter‐shot motion artifacts, outperforming conventional inter‐volume correction and demonstrating similar performance to joint estimation methods like alignedSENSE at a moderate acceleration factor (*R* = 4), with lower computational complexity. Additionally, we experimentally validated the superior motion robustness of tag‐control interleaving placed in the innermost loop. These findings highlight the potential of the proposed method to improve ASL image quality in the presence of motion, paving the way for more reliable clinical and research applications. Future work will explore higher acceleration factors and patient cohorts to further establish clinical utility.

## Funding

This work conducted in the Oxford Centre for Integrative Neuroimaging was supported by core funding from the Wellcome Trust (203139/Z/16/Z and 203139/A/16/Z) with additional support from the NIHR Oxford Biomedical Research Centre (NIHR203311) and the Oxford Health Biomedical Research Centre (NIHR203316). M.H. is supported by the Jardine Foundation. M.C. is supported by the Canada Research Chairs Program. P.J. is supported by the Vivensa Foundation and the NIHR Oxford Biomedical Research Centre. T.W.O. and J.G.W. were supported by a Sir Henry Dale Fellowship jointly funded by the Wellcome Trust and the Royal Society (220204/Z/20/Z). T.W.O. was also supported by the Podium Institute for Sports Medicine and Technology, University of Oxford.

## Conflicts of Interest

Peter Jezzard is the Editor‐in‐Chief of Magnetic Resonance in Medicine. In line with COPE guidelines, he recused himself from all involvement in the review process of this paper, which was handled by an associate editor. He and the other authors had no access to the identities of the reviewers. Frederik Lange is funded by Calico Life Sciences LLC, a subsidiary of Alphabet Inc.

## Supporting information


**Table S1:** PCC, SSIM, and tSNR values for different motion estimation and correction methods, separated by tag‐control interleaving condition. Results are averaged across all five subjects and all trajectories (CAIPI 2 × 2(1), and CAIPI 1 × 4(2)). Values are reported as mean ± standard deviation; median [min–max] across subjects. (a) T/C inner loop acquisitions. (b) T/C outer loop acquisitions.
**Figure S1:** Echo‐train simulations based on MRzero (Loktyushin et al. [31]) comparing 180°, 120°, and optimized 120° refocusing flip angle schemes. (a) Signal evolution across the spin‐echo train. (b) Corresponding PSFs in the phase‐encoding (y) and partition (z) directions, with effective resolution (EffRes) values indicated.
**Figure S2:** Navigator images reconstructed with CG‐SENSE using four regularization strengths (λ = $1\times 10^{-1}$, $1\times 10^{-2}$, $1\times 10^{-3}$, $1\times 10^{-4}$).
**Figure S3:** Coil sensitivity maps estimated via ESPIRiT from two calibration strategies: temporal average of all acquired repetitions (“All reps avg.,” left) versus average of motion‐free (MF) repetitions only (“Motion‐free reps avg.,” middle), shown for three representative coil elements (no coil compression is done in this experiment). Difference maps (right) reveal only minor discrepancies confined to peripheral voxels with low signal, with the bulk of the brain showing near‐zero difference. This confirms that full temporal averaging yields sensitivity maps essentially equivalent to those derived from a motion‐selected subset under the motion levels present in this dataset.
**Figure S4:** CG‐SENSE reconstructions from each of the four shots (*λ* = 1 × 10^−3^) for the Tag and Control repetitions, alongside the fully sampled (FS) reference. Difference maps are shown beneath each image row using a diverging colormap.
**Figure S5:** Effect of Tikhonov regularization (*λ*) on motion‐compensated SENSE reconstruction. Motion estimates were fixed across all conditions; only the regularization weight in the final MC‐SENSE reconstruction was varied.
**Figure S6:** Visualization of perfusion images for subject 3 (sagittal view). Arranged as per Figure 7.

## Data Availability

The code for the proposed motion correction pipeline can be found here: https://github.com/LoicHmh/asl_intershot_motion_correction. Data underlying the plots in the tables and figures in this paper can be found here https://doi.org/10.5281/zenodo.18152207. We are currently unable to share in vivo data due to data protection issues, although our center is actively working on a solution to this.
