# Supplementary material for: Inter‐Shot Motion Correction of Segmented 3D‐GRASE ASL Perfusion Imaging With Self‐Navigation and CAIPI
Source: Magn Reson Med. 2026 May 24;96(4):1557–71. doi: 10.1002/mrm.70437 (PMC13419355; doi:10.1002/mrm.70437)
Supplement: Supplementary file 1 — Table S1: PCC, SSIM, and tSNR values for different motion estimation and correction methods, separated by tag‐control interleaving condition. Results are averaged across all five subjects and all trajectories (CAIPI 2 × 2(1), and CAIPI 1 × 4(2)). Values are reported as mean ± standard deviation; median [min–max] across subjects. (a) T/C inner loop acquisitions. (b) T/C outer loop acquisitions. Figure S1: Echo‐train simulations based on MRzero (Loktyushin et al. [31]) comparing 180°, 120°, and optimized 120° refocusing flip angle schemes. (a) Signal evolution across the spin‐echo train. (b) Corresponding PSFs in the phase‐encoding (y) and partition (z) directions, with effective resolution (EffRes) values indicated. Figure S2: Navigator images reconstructed with CG‐SENSE using four regularization strengths (λ = 1×10^−1^, 1×10^−2^, 1×10^−3^, 1×10^−4^). Figure S3: Coil sensitivity maps estimated via ESPIRiT from two calibration strategies: temporal average of all acquired repetitions (“All reps avg.,” left) versus average of motion‐free (MF) repetitions only (“Motion‐free reps avg.,” middle), shown for three representative coil elements (no coil compression is done in this experiment). Difference maps (right) reveal only minor discrepancies confined to peripheral voxels with low signal, with the bulk of the brain showing near‐zero difference. This confirms that full temporal averaging yields sensitivity maps essentially equivalent to those derived from a motion‐selected subset under the motion levels present in this dataset. Figure S4: CG‐SENSE reconstructions from each of the four shots (λ = 1 × 10^−3^) for the Tag and Control repetitions, alongside the fully sampled (FS) reference. Difference maps are shown beneath each image row using a diverging colormap. Figure S5: Effect of Tikhonov regularization (λ) on motion‐compensated SENSE reconstruction. Motion estimates were fixed across all conditions; only the regularization weight in the final MC‐SENSE reconstruction was varied. [file MRM-96-1557-s001.docx]

**Supporting Information**

**Inter-shot Motion Correction of Segmented 3D-GRASE ASL Perfusion Imaging with Self-Navigation and CAIPI**

**Minhao Hu, Frederik J. Lange, Peter Jezzard, Joseph G. Woods, Mark Chiew and Thomas W. Okell**

## Supporting Information

***Table S1*** *PCC, SSIM, and tSNR values for different motion estimation and correction methods, separated by tag-control interleaving condition. Results are averaged across all five subjects and all trajectories ( CAIPI 2×2(1), and CAIPI 1×4(2)). Values are reported as mean ± standard deviation; median [min–max] across subjects. (a) T/C inner loop acquisitions. (b) T/C outer loop acquisitions.*


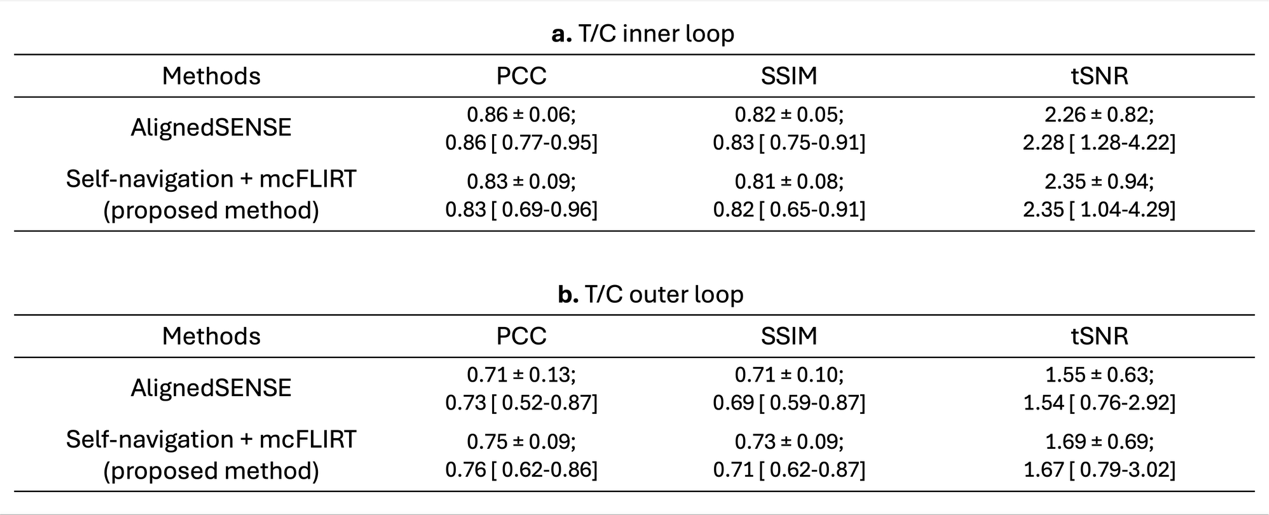


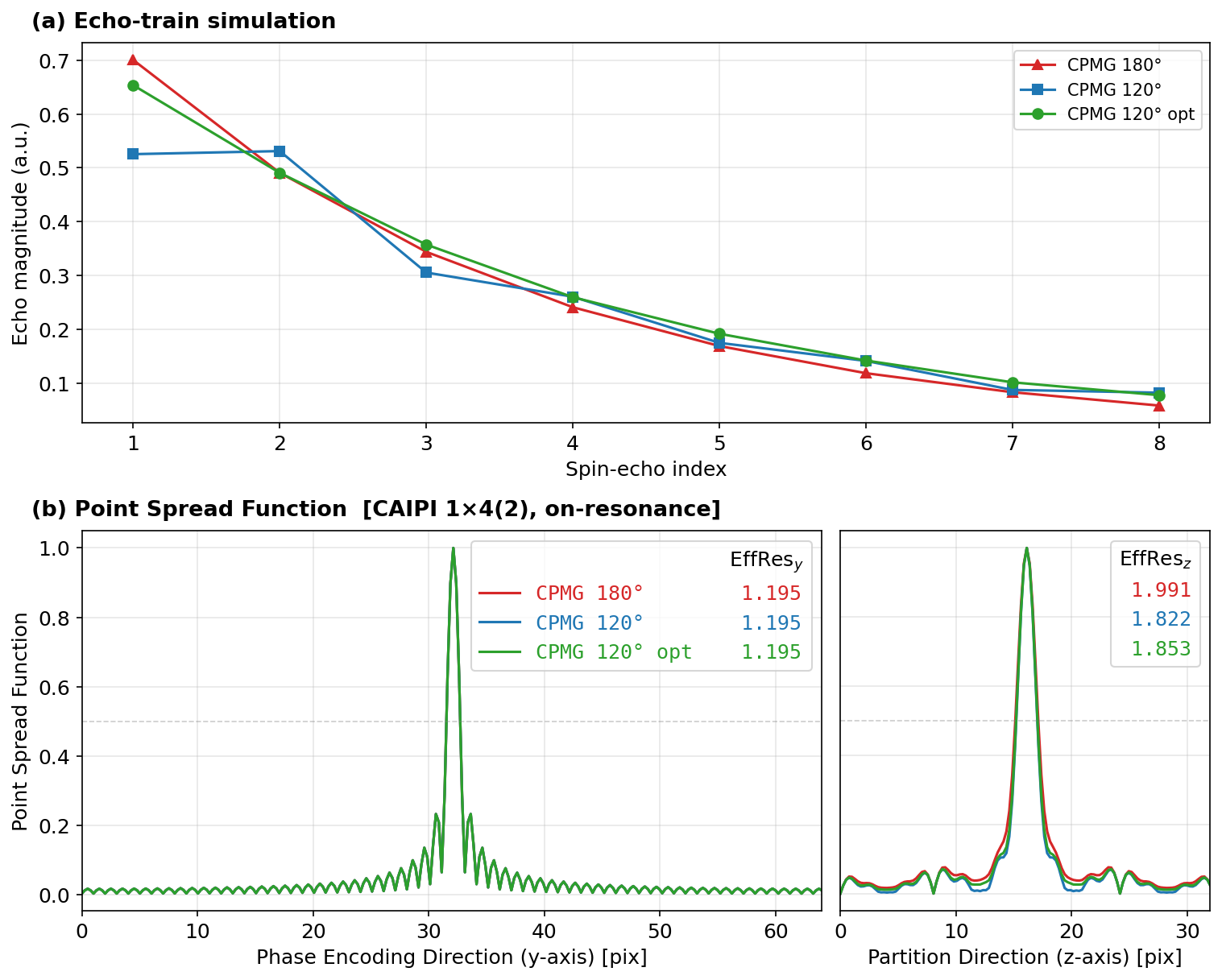


***Figure S1*** *Echo-train simulations based on MRzero (Loktyushin et al., [31]) comparing 180°, 120°, and optimized 120°^[[1]](#footnote-1)^ refocusing flip angle schemes. (a) Signal evolution across the spin-echo train. (b) Corresponding PSFs in the phase-encoding (y) and partition (z) directions, with effective resolution (EffRes) values indicated.*


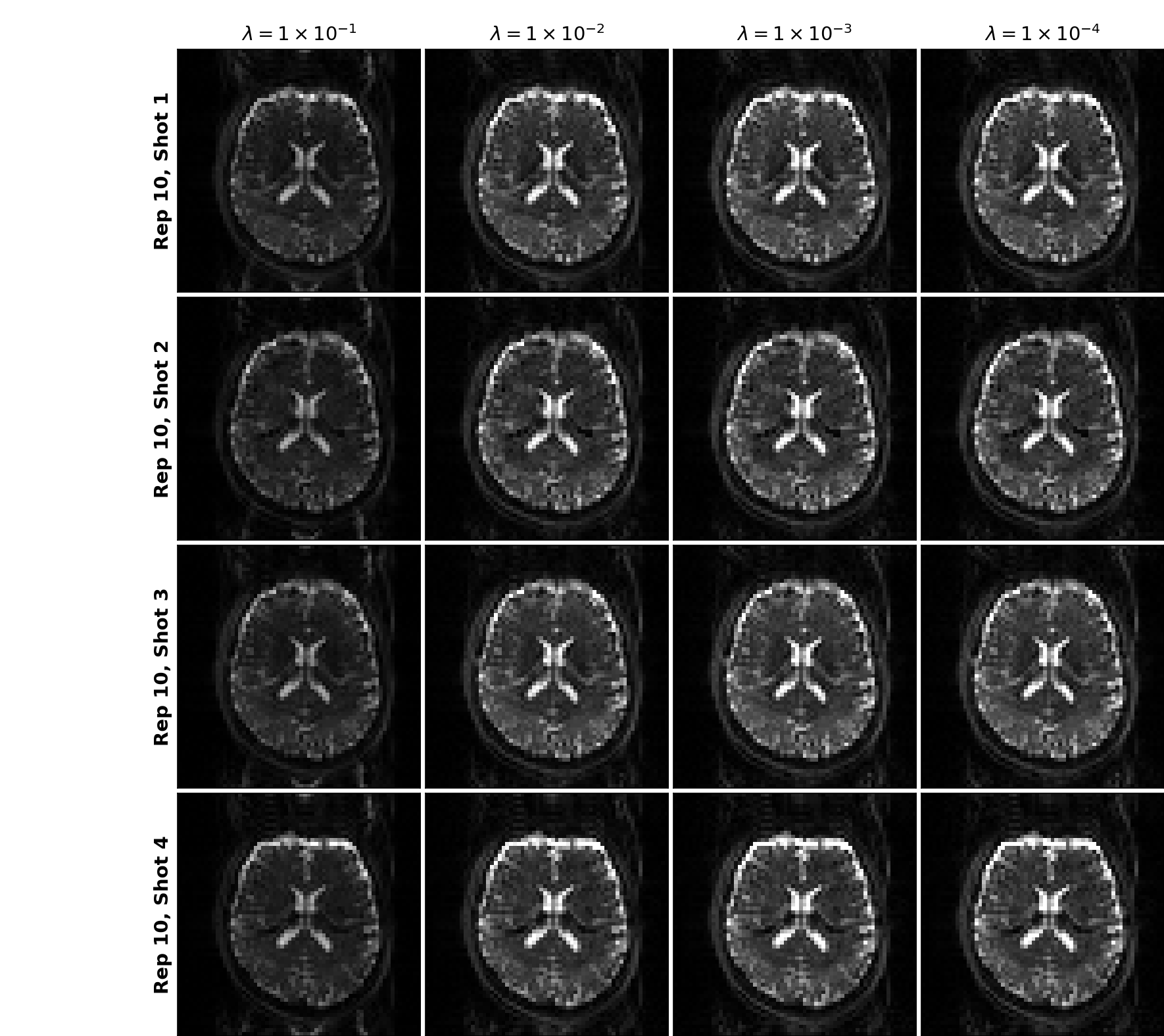


***Figure S2*** *Navigator images reconstructed with CG-SENSE using four regularization strengths (λ =* ${1\times10}^{-1}$*,* ${1\times10}^{-2}$*,* ${1\times10}^{-3}$*,* ${1\times10}^{-4}$*).*


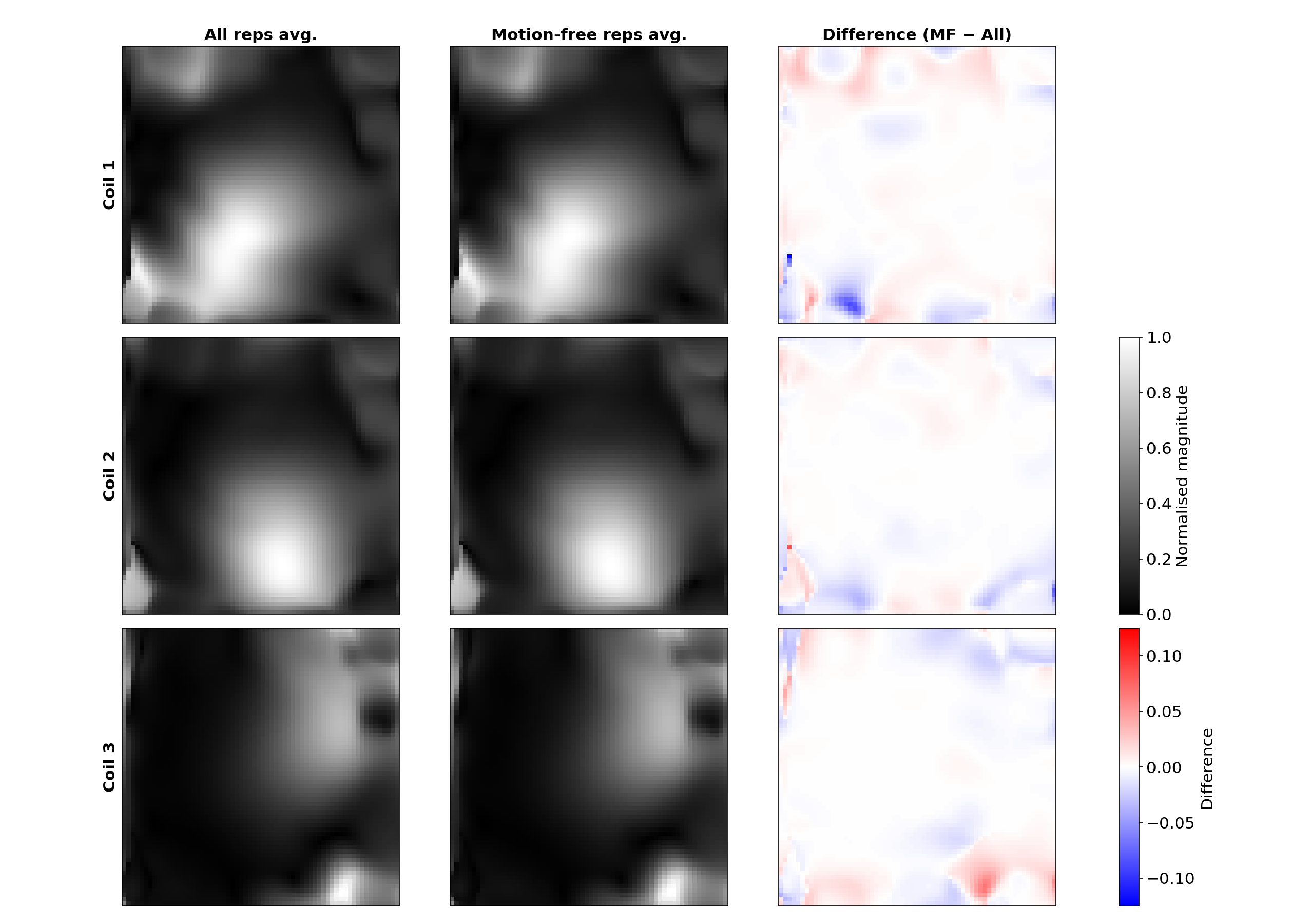


***Figure S3*** *Coil sensitivity maps estimated via ESPIRiT from two calibration strategies: temporal average of all acquired repetitions ("All reps avg.", left) versus average of motion-free (MF) repetitions only ("Motion-free reps avg.", middle), shown for three representative coil elements (no coil compression is done in this experiment). Difference maps (right) reveal only minor discrepancies confined to peripheral voxels with low signal, with the bulk of the brain showing near-zero difference. This confirms that full temporal averaging yields sensitivity maps essentially equivalent to those derived from a motion-selected subset under the motion levels present in this dataset.*


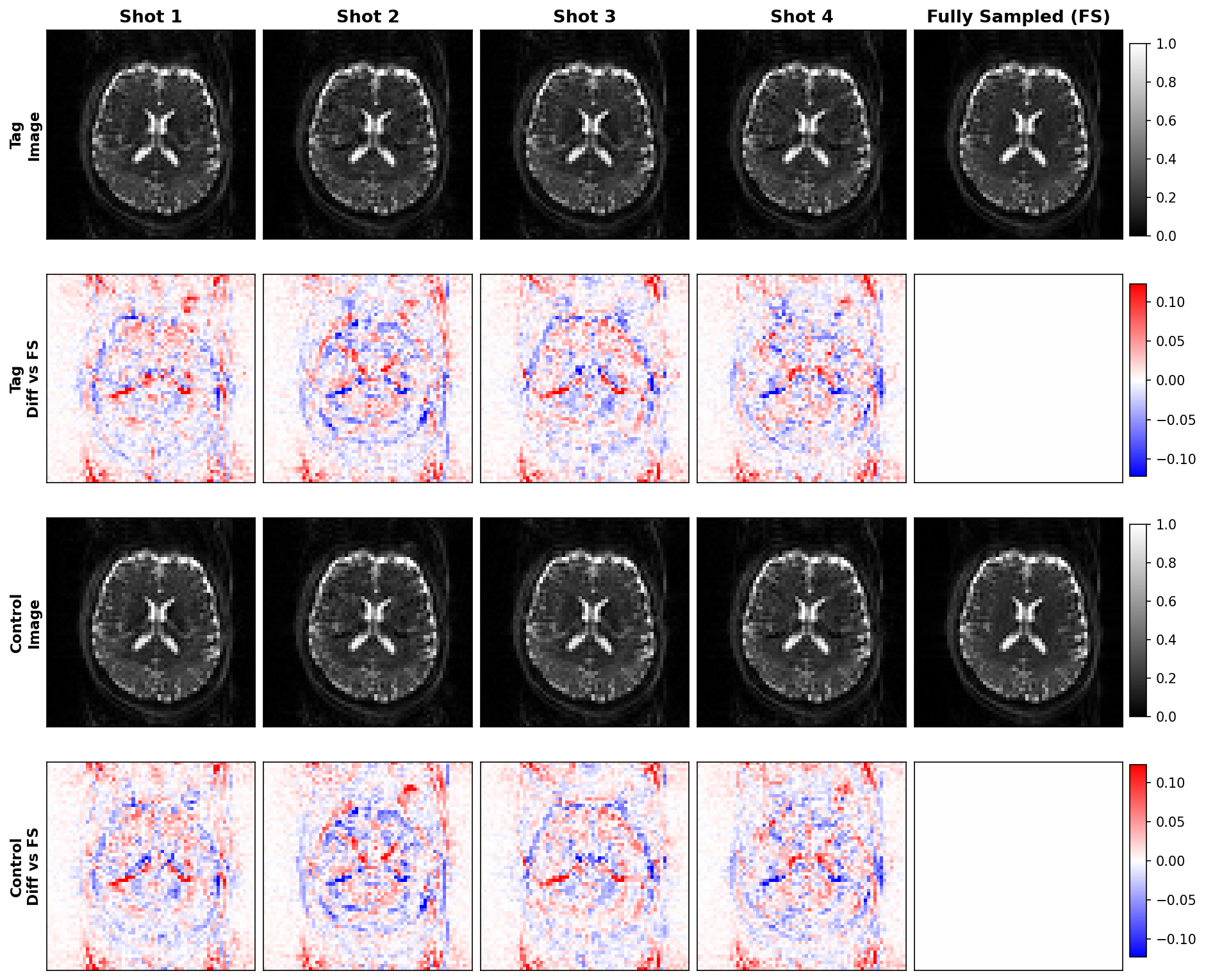


***Figure S4*** *CG-SENSE reconstructions from each of the four shots (λ = 1×10⁻³) for the Tag and Control repetitions, alongside the fully sampled (FS) reference. Difference maps are shown beneath each image row using a diverging colormap.*


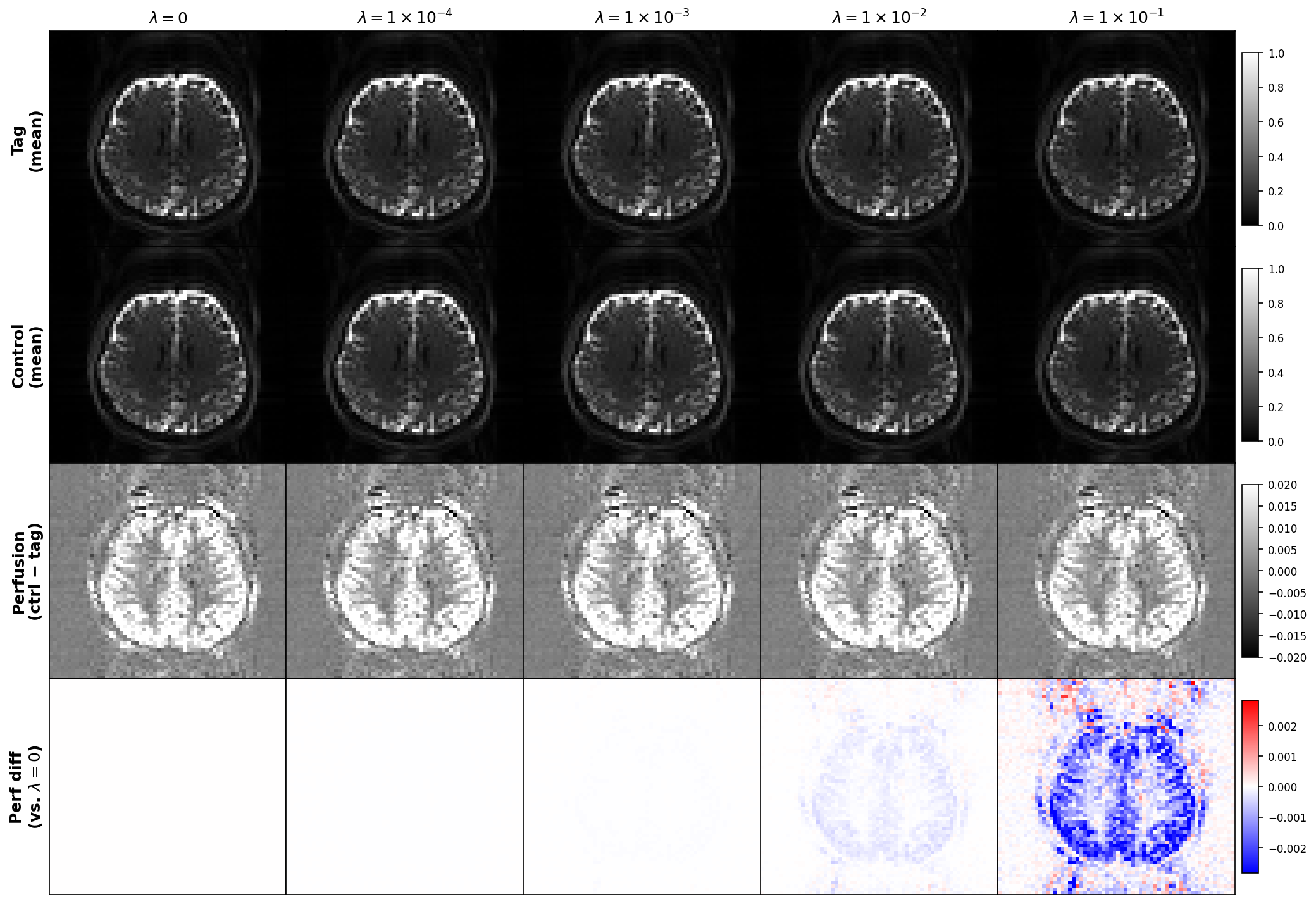


***Figure S5*** *Effect of Tikhonov regularization (λ) on motion-compensated SENSE reconstruction. Motion estimates were fixed across all conditions; only the regularization weight in the final MC-SENSE reconstruction was varied.*


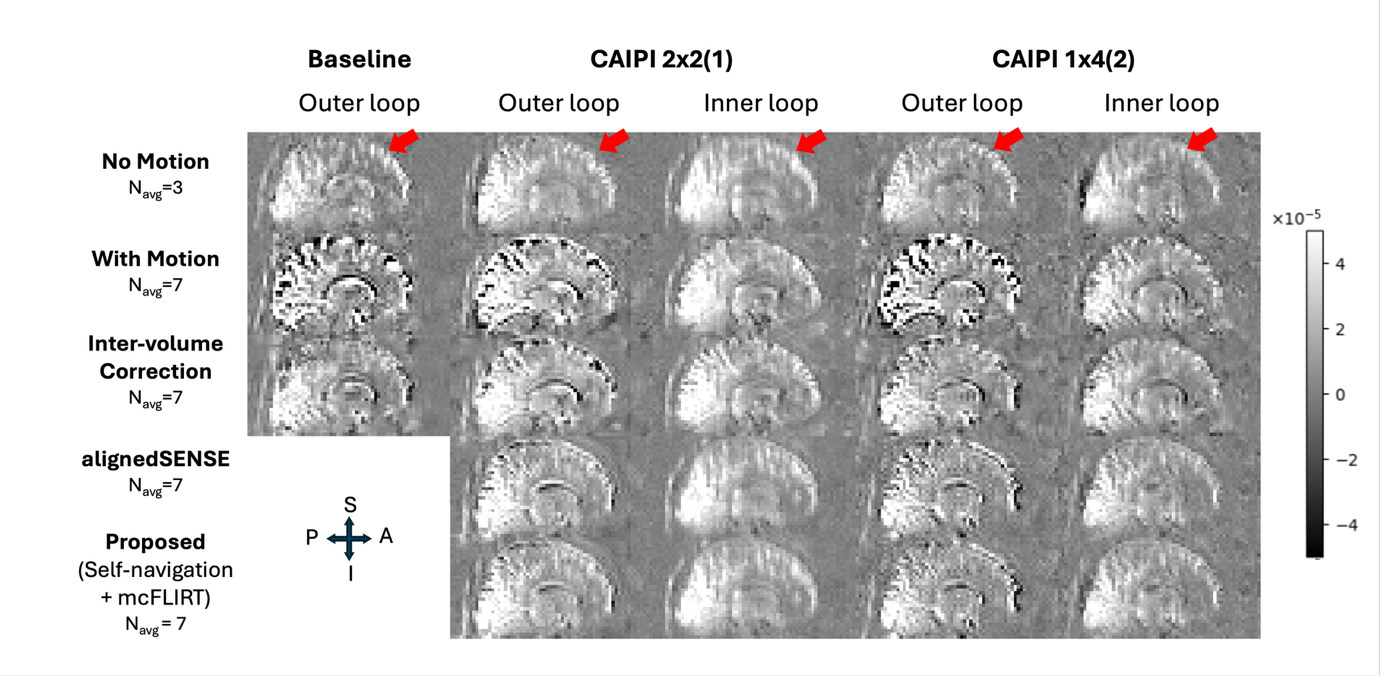


**Figure S6 Visualization of perfusion images for subject 3 (sagittal view).** Arranged as per Figure 7.

1. Hennig, J. and Scheffler, K. (2000), Easy improvement of signal-to-noise in RARE-sequences with low refocusing flip angles. Magn. Reson. Med., 44: 983-985. [https://doi.org/10.1002/1522-2594(200012)44:6<983::AID-MRM23>3.0.CO;2-8](https://doi.org/10.1002/1522-2594(200012)44:6%3c983::AID-MRM23%3e3.0.CO;2-8) [↑](#footnote-ref-1)
